# Body composition, energy expenditure and caloric intake among breast cancer patients at a teaching hospital in Nigeria—a cross sectional study

**DOI:** 10.3332/ecancer.2023.1600

**Published:** 2023-09-04

**Authors:** Ogochukwu O Izuegbuna, Toyin Sodiq, Hannah O Olawumi, Samuel A Olatoke, Olayide Agodirin

**Affiliations:** 1 Department of Haematology and Blood Transfusion, University of Ilorin Teaching Hospital, Ilorin 241102, Nigeria; 2 Dietetics Unit, University of Ilorin Teaching Hospital, Ilorin 241102, Nigeria; 3 Department of Surgery, University of Ilorin Teaching Hospital, Ilorin 241102, Nigeria

**Keywords:** body composition, energy expenditure, breast cancer, fat-free mass, dietary recall

## Abstract

**Objective:**

This cross-sectional study was conducted on the associations between body composition, energy expenditure and caloric intake among 45 Nigerian breast cancer patients.

**Methods:**

Forty-five Nigerian breast cancer patients were measured and analysed for their body composition, energy expenditure and caloric intake. Statistical analyses included a chi-square test, Student’s *t*-test, paired *t*-test, Spearman correlation and linear regression using Statistical Package for the Social Sciences 23.0.

**Results:**

The body fat indices (body mass index (BMI), fat mass index (FMI), and body fats percentage) show that more than 50% of breast cancer patients were either overweight or obese. The Spearman correlation showed that fat-free mass (FFM) was the most strongly correlated with energy expenditure (*r* = 0.84). BMI and (FMI – fat mass in relation to height) were significantly correlated with the Harris–Benedict equation for energy expenditure (*p* < 0.001; *p* = 0.002), but they were not correlated significantly with the Karnofsky performance status. A paired *t*-test showed that caloric intake was significantly higher than total energy expenditure (*p* < 0.001). FFM was the best predictor of resting energy expenditure (REE).

**Conclusion:**

In conclusion, FFM remains the best predictor of REE. High body mass and high caloric intake indicate the need for support from nutritional programmes.

## Background

Breast cancer is the most common female malignancy, and it accounted for about 30% of all cancer diagnoses in women in 2019 [1]. In Nigeria, it was estimated to be responsible for about 11,500 deaths in 2018 with an age-standardised rate of 18.8/100,000 [2]. The mortality rate of breast cancer has dipped over the past three decades in the developed world [1], but unfortunately, mortality rates are disproportionately high especially in sub-Saharan Africa [3, 4]; and with Nigeria, Africa’s most populous country having the highest mortality rate [5]. Genetic mutations are known to account for only a small percentage of breast cancer, while environmental factors play a major role in association with other factors. Due to its growing public health effects, the identification of these environmental factors as well as a screening of at-risk populations is important.

It is postulated that such environmental factors as diet and dietary intake, body composition e.g., body mass index (BMI), reduced metabolic rate and reduced physical activity are associated with breast cancer risk [6–9]. Women with higher BMI have been found to have a higher risk of mortality from breast cancer [10, 11], while animal models have provided evidence that calorie restriction can inhibit mammary tumour development [12]. An increase in dietary intake and body weight has been observed in female rats exposed to exercise training, showing an association between energy intake, expenditure and body size [13]. While BMI has long been regarded as the surrogate marker for body composition, it does not reflect true body ‘fatness’ and cannot discriminate between fat mass (FM) and lean mass [14]. This limits its usefulness especially concerning energy expenditure as fat-free mass (FFM) is known as the single best predictor of resting energy expenditure (REE) [15].

VanItallie *et al* [16] suggested the use of fat mass index (FMI) and fat-free mass index (FFMI) for a better assessment of anthropometric measurement, according to body compartments, by calculation that considers the amount of FM and FFM. The units are also expressed the same as for BMI. With their use, four types of scenarios can be identified: low FFMI and high FMI, which corresponds to obesity; low FFMI and low FMI, to leanness; high FFMI and low FMI, to muscle hypertrophy; and high FFMI and high FMI, which correspond to combined excess of FFMI and FMI. However, their reference range is not yet a consensus in scientific works of literature.

On the other hand, REE (which is the largest component of total energy expenditure (TEE)) is known to be variable in cancer, and it is influenced by several factors including body composition [17]. Furthermore, reduced energy expenditure plays an important role in the development of obesity by decreasing REE, energy activity, diet-induced thermogenesis or a combination of all of these components [14]. Thus, an increase in caloric intake with energy expenditure as well as body composition may be a risk factor for breast cancer. The objective of this study was to evaluate the body composition and REE of breast cancer patients as well as assess the association between dietary intake, as a function of energetic expenditure and energy requirements.

## Materials and methods

This cross-sectional study was conducted at the University of Ilorin Teaching Hospital, Ilorin, from January 2018 to June 2018. Ethical approval was obtained from the Hospital’s Ethical Research Committee (ERC PAN/2015/12/1483). The study group comprised 45 histologically diagnosed female breast cancer patients who are >18 years old. Informed consent was obtained from each participant. The exclusion criteria included patients who were pregnant/breastfeeding, diagnosed with any other cancer, uncontrolled chronic comorbidities such as hypo/hyperthyroidism, on anti-hypertensive, on lipid-lowering drugs (HMG CoA reductase inhibitors (statins), bile acid sequestrants, nicotinic acid, fibric acids), on hormonal medications, with a Karnofsky performance status less than 50%, hospitalised or bedridden and patients with apparent nutritional impairment were excluded from this study. The patients were staged clinically according to the tumour, node and metastasis classification.

### Height/weight

The participants’ weight was measured in kilograms (kg) using a bathroom scale with participants wearing minimal clothing and no shoes. Height was measured in meters using a stadiometer with the participants standing erect, bare-footed and looking straight ahead.

### Body composition

The BMI was derived as weight divided by height squared expressed as kg/m^2^. A proprietary algorithm for calculating body fat percentage (BF%) was used. The equation of Deurenberg et al [18] was used to calculate the BF%. The FM was derived as a product of the BF% and body weight. FFM was determined through the formula: weight in kg × (1 − (BF%/100)). FMI and FFMI were determined by dividing the FM and the FFM by the square of the height as proposed by VanItallie *et al* [16]. The BMI was categorised as 18.5–24.9 kg/m^2^ as normal, 25–29.9 kg/m^2^ as overweight and >30 kg/m^2^ as obese, while the BF% was classified as <23% underfat, 23%–33% healthy, 33%–39% overweight, 40% and above as obese. The categorisation of the FMI was <5 as underweight, 5–9 as normal, 9–13 as overweight and >13 as obese, while FFMI was <18 skinny, 18–20 average, 20–22 good and >22 excellent.

### Energy expenditure

The REE predictive equation used in this study was the Harris–Benedict equation (HBE) [19]. The TEE was estimated by multiplying the REE calculated from HBE with the activity factor (AF) and the injury factor (IF).

TEE (from HBE) = REE (HBE) × AF × IF where AF = 1.3 and IF = 1.1

The energy requirement was further estimated using the 25–30 kcal/kg/day formula.

### Dietary intake

Because dietary intake is a key component of energy balance, dietary intake was assessed using the 48 hours of dietary recall. The type and amount of food that the patient consumed in the last 48 hours were asked by a trained nutritionist. All recalls were analysed with the Nigerian Food Composition table version 1.0 [20].

The physical and functional ability were determined using the Karnofsky performance status scale.

### Statistical analysis

Statistical analysis was performed using Statistical Package for the Social Sciences version 23.0. The descriptive data were given as mean ± SD. A correlation analysis was conducted to see the correlation between the parameters using the Spearman correlation analysis. The *R*-values were obtained to observe the strength and direction of the correlation. A paired *T*-test was performed to compare the mean difference between the 48 hours of dietary recall and TEE. Predictors of REE were evaluated using multiple linear regression analysis with REE as the dependent variable and body composition as the independent variables.

## Results

**Figure 1.**
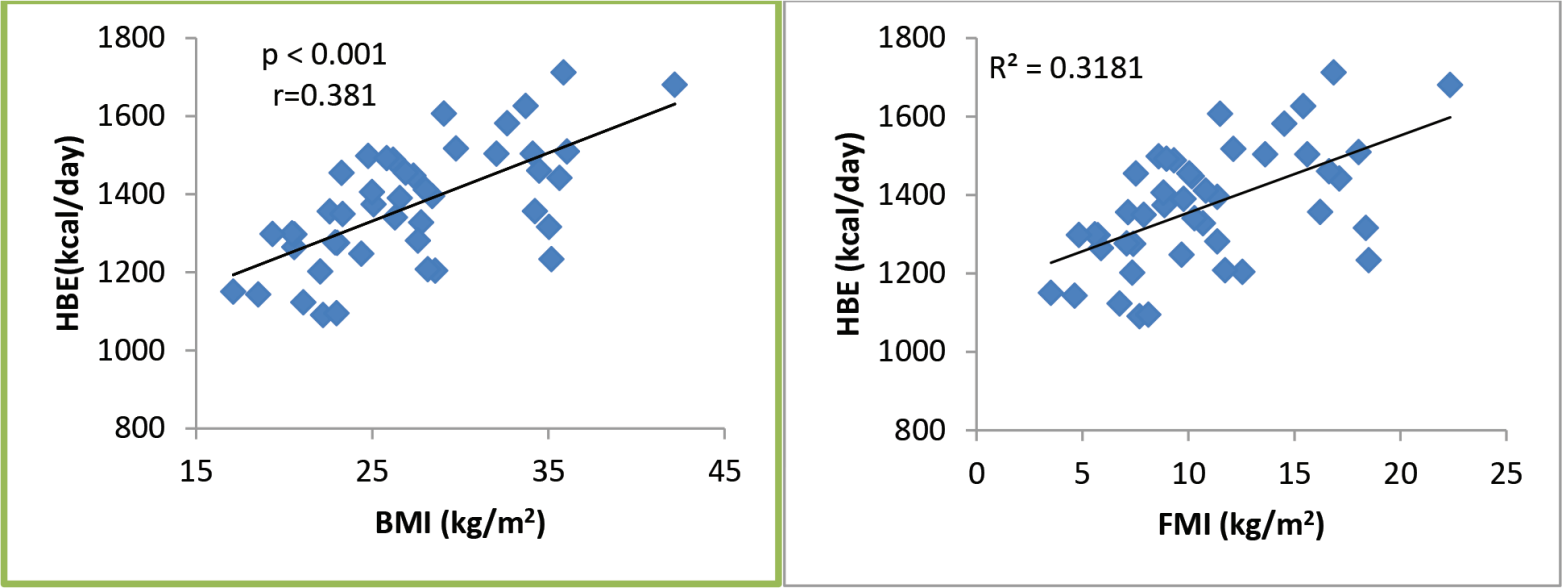
REE as related to BMI and FMI in 45 breast cancer patients. All correlations are significant.

**Figure 2.**
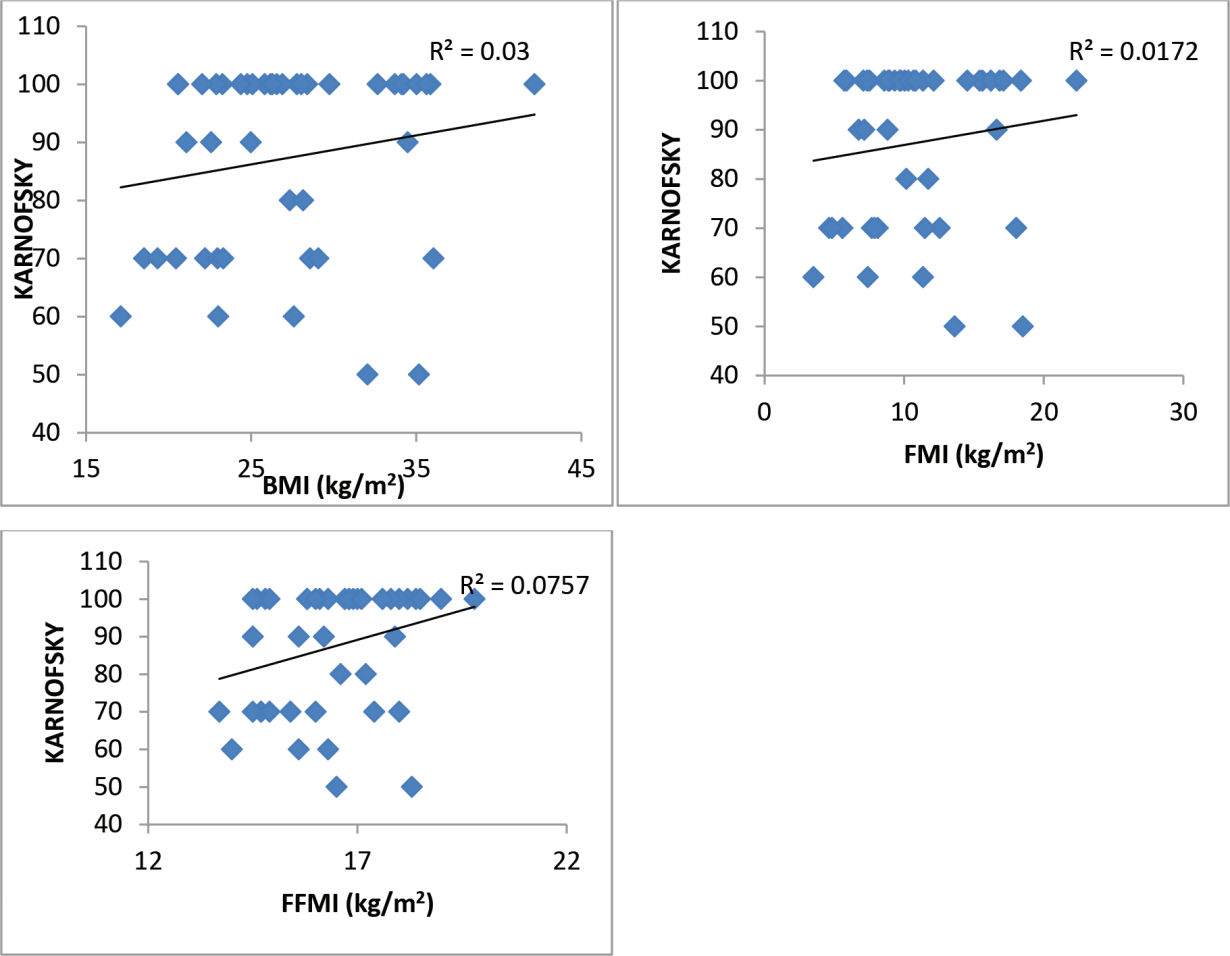
Karnofsky performance status as related to BMI, FMI and FFMI in 45 breast cancer patients. Only the FFMI correlation is significant.

A total of 45 female breast cancer patients were included in this study. The mean patient age was 49.3 ± 14.8 years. The anthropometric characteristics and predicted energy requirement of the participants are presented in Table 1. The predicted REE which was the HBE positively correlated significantly with all components of body composition. Additionally, the ratios of HBE/FFM and HBE/BW were negatively correlated significantly as shown in Table 2. While BMI and FMI were significantly correlated with HBE, they were not correlated significantly with the Karnofsky performance status as shown in Figures 1 and 2.

Table 3 shows the energy requirements of the study with their daily caloric intake as well as the expected intake using a fixed amount of 25–30 kcal/kg of BW. The 48 hours recall showed a difference of 263 kcal compared to the TEE derived from the HB equation multiplied by the activity and the IF. Using the fixed amount of 25–30 kcal/kg of BW (i.e., 29 kcal/kg) a minor difference of 61 kcal was underestimated. Furthermore, the fixed amount of 25–30 kcal/kg of BW was more consistent with the TEE as there was no significant difference between both values.

The multiple linear regression analysis of REE was used to relate REE to the body composition variables. FFM was the single best predictor of REE among female Nigerian breast cancer patients in this study as shown in Table 4.

## Discussion

This study was embarked on to observe the body composition as well as the energy expenditure and its predictors among Nigerian breast cancer patients which have not been reported before now. The mean age (49.3 ± 14.8 years) of the study sample indicates breast cancer continues to be a disease of the middle-aged and younger age group (<40 years) in this part of the world [21].

The association between body fat and cancer risk has been noted in different studies [22, 23]. Traditionally, body fats are determined through the BMI which is known as a poor differentiator of lean mass and FM. On the other hand, FMI is a better marker of adiposity and obesity than BMI [24], although this was in Mexican Americans. However, BMI is strongly correlated with FMI and body fats according to a study carried out by Jeong *et al* [25], though such issues as body composition are linked to race and ethnicity [26]. Like in previous studies [27, 28], our study also showed that BMI is strongly correlated to FMI and body fats. However, while there is a similarity between BMI and FMI from our data, body fats estimated more people as being obese than BMI, the clinical significance is undetermined.

Body fat distribution is a well-known predictor of cardiovascular disease [29], some cancers [30, 31] and mortality [32, 33], thus accurate estimation of body fats is important in the management of obesity-related conditions. Equally, a meta-analysis of 221 datasets showed that the risk ratio for breast cancer incidence increased by 1.12 for every 5 kg/m^2^ increase in BMI for postmenopausal women [34]. *In lieu* of the aforementioned risks, our breast cancer patients in this study may need a weight management plan to prevent some morbidities.

The REE of women with breast cancer was determined using the HBE, which has been shown to give a higher value of REE compared to indirect calorimetry [35]. However, HBE and REE/BW were significantly correlated to the different body compositions showing that body compositions contribute to REE.

Individuals with cancer experience signiﬁcant changes in body composition and REE with varying factors such as oral intake, physical activity, inflammatory processes, etc. REE is noted to be a result of metabolic activities within body tissues and organs especially organs like the liver and lungs. Elevated REE can potentially promote weight loss which can produce suboptimal clinical outcomes and increase morbidity and mortality risk. A relationship between REE, cachexia and survival were observed by Vazeille *et al* [36]. They also found that hypermetabolism, but not hypometabolism, was associated with reduced survival in metastatic cancer. Our study, however, did not evaluate survival with respect to levels of energy expenditure. It, however, showed that energy expenditure is significantly positively correlated with the various body composition indices with FFM having the strongest correlation as shown in Table 2. While tumour burden has been proposed to affect energy expenditure, our study did not show any significant correlation between REE and stage, tumour size and lymph node status. It, however, was significantly correlated with performance status.

The role of energy intake on breast cancer risk has been studied in both experimental and observational studies. According to the International Agency for Research on Cancer, there is sufficient evidence from experimental animal studies indicating that limiting body-weight gain by caloric restriction has a preventive effect on cancer of the mammary gland [30]. Energy intake, energy expenditure and body composition are three factors that are known to determine energy balance, and in turn, affect several biological pathways involved in cancer initiation. Caloric restriction is associated with decreased circulating levels of growth factors, anabolic hormones and cytokines which in turn lead to a reduction in growth factor signalling, vascular perturbations and inflammation. On the other hand, high energy intake has not been consistently associated with increased breast cancer risk in human studies [37]. Thus, a positive energy balance characterised by excess energy intake (nutritional intake) with little physical activity over a prolonged period, results in excess body fat and, consequently, contributes to increased postmenopausal breast cancer risk.

This present study also analysed the association between relative energy intake (dietary recall), and breast cancer with TEE and expected caloric estimate (25–30 kcal/kg BW). Our result shows that there is a positive energy balance with energy expenditure, and a caloric estimate of 29 kcal/kg BW is sufficient to meet daily requirements. This positive energy balance is also statistically significant thus the excess body fats in our subjects may be a result of nutritional intake. Lope *et al* [38] in the EPIGEICAM study, were able to show that high energy intake increases breast cancer risk, and caloric restriction could be protective.

While excess fats are a breast cancer risk, especially in postmenopausal women, it should be noted that FM contributes to REE. FFM is the single best predictor of REE [15] as also noted in our study; both FFM and FM were predictors of REE. Interestingly while FFM has the strongest positive correlation with REE in our study (*r* = 0.84; *p* < 0.01), it has the weakest negative correlation with HBE/kg of BW, while FM has the strongest negative correlation with HBE/kg of BW (*r* = −0.92; *p* < 0.01). In healthy adults, a decrease in FFM explains approximately 60% of the decrease in REE observed with age [39]. However, FFM is known to be a heterogeneous body composition compartment that comprises tissues with differing metabolic activity thus in metastatic cancer, it is unable to distinguish between changes in tissue, organs and tumours. A highly metabolic organ like the liver will be expected to have a greater REE impact in metastatic disease to the liver than on other less metabolic organs. Thus, two individuals with the same disease stage may not necessarily have the same REE. In our study, overall REE decreased in patients with stage III disease and increased in patients with stage IV disease (data not shown), which could be suggestive of extra tumour burden. This was also reported by Purcell *et al* [17] in colorectal cancer patients. In the correlation test, both BMI and FMI were positively correlated with REE (Figure 1). This may be because obese people tend to have increased FFM to probably support the FM. Nielsen *et al* [15] also reported the predictive significance of FFM and FM in REE, although their study did not include breast cancer patients.

In summary, our study shows that excess body fats among other variables in our breast cancer patients may explain their breast cancer status. The excess body fats may also be a result of increased caloric intake as derived from their 48 hours of dietary recall. This is the first study in Nigerian breast cancer patients that examine their body composition in association with energy expenditure as well as caloric intake. It thus brings to the fore the need for public health awareness of overweight and obesity and its association with the increasing numbers of breast cancer cases in Nigerian society. The increased consumption of fast foods in the country [40] also contributes to the obesity epidemic and high caloric intake. It is reported that what contributes to obesity and related diseases is not the number of calories in specific foods but rather the amount and type of carbohydrates these foods contain. Foods that are highly processed, and mostly comprised of rapidly absorbable sugars and starches, may be of greatest concern. Such carbohydrates may induce neurohormonal alterations that lead to ‘eating more’ and ‘moving less’ [38]. Individual and public health strategies should thus be implemented to help curb it. Caloric restriction may play a beneficial role in health and well-being. Finally, simple measures of body fat distribution and body composition have been found to be useful tools for identifying Black women with a higher risk of death after a breast cancer diagnosis. Bandera *et al* [41] very recently reported that higher adiposity was associated with higher all-cause and breast cancer-specific mortality among Black breast cancer survivors. Weight gain rather than weight loss is reported to occur in breast cancer patients especially during chemotherapy [42]. It is postulated that this weight gain is related to changes in caloric intake, reduced physical activity and metabolic rate and hormonal changes [6]. This weight gain increases the likelihood of complications, increases the risk of recurrence and results in a low survival rate [43].

Based on the results of this study, we propose that a nutrition planning/weight control programme and physical workout plan should be part of the supportive care of Nigerian breast cancer patients.

## Limitations

Several limitations should be considered when interpreting our findings. First, it is a single-centre, cross-sectional study. A multi-centre prospective study would need to be carried out to further confirm our findings. Second, the small number of our study group may increase bias. A larger multi-centre study group may be needed to validate our results. Third, due to cost and limited expertise, most patients did not carry out immunohistochemistry tests such as oestrogen, progesterone receptors, HER-2 and Ki-67, so they could not be reported. While indirect calorimetry is the gold standard for measuring REE, due to non-availability at our centre, logistics and its impractical nature for clinical use could not be assessed for this study. Indirect calorimetry is expensive, time-consuming and requires trained technicians, and to the best of our knowledge is not available in our country so the need to use a predictive equation. Body composition especially body fats could not be assessed by a bioelectrical impedance analyser or dual-energy X-ray absorptiometry due to non-availability at our centre, we, therefore, used anthropometric equations to derive our body composition. The self-reported dietary recall is subject to bias based on the extent of recall of diets taken during the period examined.

This study equally has its strength. First, this is the first study to examine the body composition among breast cancer patients in Nigeria, most importantly looking at other indices other than BMI e.g., FMI and FFMI. Second, to our knowledge, this is the first study on energy expenditure in Nigerian breast cancer patients and its association with daily energy requirements (25–30 kcal/kg BW) and excessive caloric intake. Third, we believe our results from this study may spur other research in the study of body composition, energy expenditure and well-being of the Nigerian breast cancer patient.

## Conclusion

In conclusion, the breast cancer patients in this study have excess fat. The caloric intake of breast cancer patients was significantly higher than their TEE. The importance of this is the need to set up a nutritional programme for breast cancer patients and monitor their calorie intake. This can help in the reduction of excess fats and help promote healthy lifestyles.

## Conflicts of interest

No potential conflict of interest was reported by the authors.

## Funding

The researchers did not receive any external funding for this work.

## Data availability statement

The data that support the findings of this study are available from the corresponding author, (OI), upon reasonable request.

## Author contributions

OI was involved in the design of the study, data analysis and writing of the first draft manuscript. TS was involved in data analysis, study coordination and correction of the draft. SO, HO and OA were involved in the design of the study, coordination and corrections of the draft manuscript. All authors read and approved the final manuscript.

## Figures and Tables

**Table 1.** Socio-demographic and anthropometric characteristics of study group.

Age (year) <40 41–60 ≥61 Mean ± SD	17 (38.7) 19 (42.2) 9 (20.0) 49.26 ± 14.85
Level of education None Primary Secondary Tertiary	10 (22.2) 6 (13.3) 11 (24.4) 18 (40.0)
Histologic type Invasive ductal Lobular	43 (95.6) 2 (4.4)
Stage (*n*, %) 1 2 3 4	2 (4.4) 10 (22.2) 22 (48.9) 11 (24.4)
BMI (kg/m^2^) Underweight Normal Overweight Obese Mean ± SD	1 (2.2) 17 (37.8) 15 (33.3) 12 (26.7) 27.25 ± 5.70
Body fat (%) <23 24–33 34–39 ≥40 Mean ± SD	1 (2.2) 13 (28.9) 13 (28.9) 18 (40.0) 38.22 ± 7.80
FMI (kg/m^2^) Underweight Normal Overweight Obese Mean ± SD	3 (6.7) 16 (35.6) 14 (31.1) 12 (26.7) 10.82 ± 4.38
FFMI (kg/m^2^) <18 18–19.9 20–21.9 ≥22 Mean ± SD	37 (82.2) 8 (17.8) 0 (0.0) 0 (0.0) 16.42 ± 1.43
REE (HBE) TEE REE/kg FFM REE/kg BW	1,370.9 ± 152.9 2,073.6 ± 210.2 32.9 ± 2.0 20.2 ± 2.6

BMI – body mass index; FMI – fat mass index; FFMI – free-fat mass index; REE – resting energy expenditure; TEE – total energy expenditure

**Table 2.** Spearman correlation of energy expenditures and body composition in breast cancer patients.

	HBE		HBE/FFM		HBE/BW	
	*r*	*p*-value	*r*	*p*-value	*r*	*p*-value
Body fat (%)	0.24	0.018	−0.12	0.239	−0.84	<0.001
BMI (kg/m^2^)	0.38	<0.001	0.02	0.889	−0.88	<0.001
FM (kg)	0.50	<0.001	−0.11	0.303	−0.92	<0.001
FFM (kg)	0.84	<0.001	−0.66	<0.001	−0.44	<0.001
FMI (kg/m^2^)	0.31	0.002	0.05	0.642	−0.91	<0.001
FFMI (kg/m^2^)	0.53	<0.001	0.02	0.86	−0.73	<0.001

BMI – body mass index; FMI – fat mass index; FFMI – free-fat mass index; HBE – Harris–Benedict equation; FFM – free-fat mass; FM – fat mass

**Table 3.** TEE in women with breast cancer (*n* = 45).

Method	TEE (kcal/day)	Mean difference	95% CI	*p*-value
TEE 48 hours recall 29 kcal/kg BW	2,073.6 2,336.6 2,012.6	−263.0 −61.0	−371.8; −154.1 −131.9; 9.8	<0.001 0.090

TEE – total energy expenditure

**Table 4.** Multiple linear regression analysis with the REE as a dependent variable.

Variables	*b*	95% CI	Partial *r*^2^	*p*-value
BF% BMI FMI FFMI FM FFM	58.3 87.0 72.4 64.4 8.5 20.8	28.9–87.7 51.2–122.9 43.7–101.0 23.1–105.8 6.0–10.9 17.9–23.7	0.13 0.25 0.20 0.08 0.33 0.68	<0.0001 <0.0001 <0.0001 0.003 <0.0001 <0.0001

BMI – body mass index; FMI – fat mass index; FFMI – free-fat mass index; BF% – body fat percentage; FFM – free-fat mass; FM – fat mass
